# Behavioural Effects and Market Dynamics in Field and Laboratory Experimental Asset Markets

**DOI:** 10.3390/e22101183

**Published:** 2020-10-20

**Authors:** Sandra Andraszewicz, Ke Wu, Didier Sornette

**Affiliations:** 1 Chair of Cognitive Science, Department of Humanities, Social and Political Sciences (D-GESS), ETH Zurich, 8092 Zurich, Switzerland; 2 Institute of Risk Analysis, Prediction and Management (Risks-X), Academy for Advanced Interdiscplinary Studies, Southern University of Science and Technology (SUSTech), Shenzhen 518055, China; 3 Chair of Entrepreneurial Risks, Department of Management, Technology and Economics (D-MTEC), ETH Zurich, 8092 Zurich, Switzerland; 4 Swiss Finance Institute, c/o University of Geneva, 1211 Geneva, Switzerland

**Keywords:** experimental asset markets, psychological entropy, replicability, laboratory experiments, field experiments, complex systems

## Abstract

A vast literature investigating behavioural underpinnings of financial bubbles and crashes relies on laboratory experiments. However, it is not yet clear how findings generated in a highly artificial environment relate to the human behaviour in the wild. It is of concern that the laboratory setting may create a confound variable that impacts the experimental results. To explore the similarities and differences between human behaviour in the laboratory environment and in a realistic natural setting, with the same type of participants, we translate a field study conducted by reference (Sornette, D.; et al. *Econ. E-J.* **2020**, *14*, 1–53) with trading rounds each lasting six full days to a laboratory experiment lasting two hours. The laboratory experiment replicates the key findings from the field study but we observe substantial differences in the market dynamics between the two settings. The replication of the results in the two distinct settings indicates that relaxing some of the laboratory control does not corrupt the main findings, while at the same time it offers several advantages such as the possibility to increase the number of participants interacting with each other at the same time and the number of traded securities. These findings pose important insights for future experiments investigating human behaviour in complex systems.

## 1. Introduction

Financial markets are social systems characterised by self-organising structure and emergence of critical events such as bubbles and crashes under conditions of persistent uncertainty. Due to the high complexity of financial markets, it is difficult to disentangle the interaction among individually measured variables from random or not-measured variables. Therefore, most of existing studies are simplified to a highly controlled laboratory setting [1]. However, it is not clear whether uncertain environments created in these markets result in levels of entropy comparable to real markets and how experimental conditions impact psychological entropy of agents in these experiments.

*Psychological entropy* is a model of uncertainty defined as “the experience of conflicting perceptual and behavioural affordances” [2]. In agreement with Darwin’s evolutionary perspective and Freud’s psychological-clinical view, anxiety is a feeling of apprehension, tension, nervousness and worry, often accompanied with physiological arousal, such as stress [3]. From the cognitive science perspective, “uncertainty is experienced subjectively as emotion of anxiety and is associated with activity in the anterior cingulate cortex and heightened noradrenaline release” [2]. It is important to note that emotion of anxiety should be distinguished from the emotions of fear and the personality trait-anxiety. A vast amount of research in cognitive psychology indicates that anxious individuals interpret ambiguous situations more negatively and interpret them as more threatening (see [4] for a representative review). Anxiety can induce attention bias such that ambiguous stimuli can be interpreted as more negative and possible negative consequences of choice outcomes can be seen as more likely than the positive consequences [5]. In addition, affect including anxiety has been shown to play an important role in inflating bubbles in experimental asset markets [6].

Another strain of research demonstrates that context in which one participates in an experimental asset market can impact mispricing on the market. For example, Reference [7] demonstrated that higher fraction of women in experimental asset markets leads to lower mispricing (less pronounced bubbles). While the authors attribute this effect to gender differences in price expectations, competitiveness and risk-taking, it is not clear how persistent these gender differences would be if participants were not aware of the gender of other market players. Despite the fact that a large number of laboratory studies with naïve students and financially untrained participants demonstrate higher risk preferences in men than in women, studies with professional managers and entrepreneurs show no gender differences in risk preferences [8]. Research in judgement and decision making highlights that context of a task determines used strategies [9,10]. More familiar choice options evoke different valuation of these alternatives by decision makers [11]. All these empirical findings are in line with the notion of *social entropy* [12] assuming that a society is a system that can be at different states of order, where even a well-ordered society has to deal with novel events stemming from the environment. However, research on the impact of the order in the society and external stimuli influencing this order on psychological entropy has rarely utilised experiments with human subjects.

Experimentally studying psychological entropy in complex systems such as asset markets poses a number of challenges. In our opinion, the biggest challenge is that real asset markets offer a large number of securities to a large number of market participants who can interact with each other at various times during trading hours. The interaction can happen over buy and sell orders and through interpersonal communications. The laboratory setting reproduces these features in a very limited and reductionist way while, on the other hand, reducing possible effect of uncontrolled variables. The trade-off between internal and external validity of psychological experiments is important and difficult to address when investigating human behaviour in complex systems such as financial markets. This is because decisions made at an early stage of the experiment and interactions between individual persons can result in further bifurcations and path-dependencies at later stages of the experiment [13,14].

Therefore, the aim of this paper is to investigate the impact of complexity of experimentation environment on replicability and generalisability of behavioural effects and market dynamics in experimental asset markets. Following [15], replicability of a system “is in large part concerned with the information and patterns within a system as the core item of interest, rather than particular physical expressions”. Following this logic, an effect is replicable, not when it is identical to its original version, but when it is characterised by the same features as the original, even when the system is in a different state than the original one. In behavioural science terms, this would mean that a behavioural effect is replicated when it occurs in similar, but slightly altered experimental conditions (i.e., laboratory vs. field).

In this paper, we first test whether we find the same behavioural effects in the field and in the laboratory. In addition, we aim to investigate the dynamics of the two types of experimental markets populated by the same type of participants, in order to assess the impact of the environment on their behaviour. Finally, we aimed to close the gap on the field-lab comparison for experimental asset markets with multiple securities. For this purpose, we replicate in the laboratory the framed field study [16] conducted by [17], using the same experimental material and the same type of participants. The design of [17] is sufficiently engaging as a field study conducted over a few days, while being simple enough to be run within one experimental session. This property allows for testing the impact of experimentation in the artificial laboratory environment on the experimental results and behavioural dynamics of the study participants.

## 2. Laboratory and Field Economic Experiments—A Literature Review

While laboratory studies allow for controlling the variables of interest, they are often open to the criticism that their environment is quite artificial [18]. Authors in [19] argue that lack of realistic conditions is not a problem and that laboratory markets are real markets as long as the general economic principles apply. According to this reasoning, artificiality is not an issue if an experiment allows for testing and comparing particular theories [1]. However, in [20] the author points out that highly structured markets, such as those implemented in laboratory experiments, are rare in real life. He indicates that “most of the economic transactions [...] are notable for the lack of disciplining mechanisms.” Therefore, “laboratory experiments are of limited relevance for predicting field behaviour, unless one wants to insist a priori that those aspects of economic behaviour under study are perfectly general” [16]. Moreover, the control in the laboratory may paradoxically introduce unintended variables that are not present in the wild, such as limited time, lack of field-specific knowledge, etc.

One way of investigating the robustness of experimental results is their replicability. Repeating the experiments is a way to define whether the particular finding is a true stylised fact or rather an artifact generated by inexperience, coincidence or mistake [21]. The issue of replicability in behavioural sciences has been addressed by [22], who have replicated 100 original studies published in three top journals in psychology. Following this tradition, Reference [23] replicated 18 studies in the American Economic Review and the Quarterly Journal of Economics. In [22] they reported reproducibility of 36%, while [23] reported that the results were replicated in 61% of the studies.

The need for replicability of results is reflected by the creation of electronic libraries of standard experimental tasks [24]. However, the fact that a particular effect is replicated many times in a very similar setting does not imply that this effect is of any relevance outside of this environment. Following this line of reasoning, one could fall into a trap of testing theories in an isolated environment that hold under the assumptions of this environment [18]. Note, that in the “classical” economic research, theories would be proven by mathematical derivation, ignoring anomalies in the data and variables not considered by the model. Analogically, experimental economists may fall prey to making the same mistake by ignoring important experimental methodological issues related to artificiality of the experimental setting.

Our approach to replicability of experimental results is different—we aimed to evaluate the generalisability of behavioural effects obtained both in more realistic and in artificial experimental environments. Towards this goal, we translate an experimental asset market study that was conducted in the field to the laboratory setting. We use the same experimental material and rules, but we adapt the procedure to the sterile laboratory environment. The point of this exercise is to challenge a frequent misconception about field studies that field experiments are the “uncontrolled variants of laboratory experiments” [25]. On the contrary, we propose that the domain of experimental asset markets conducted in the laboratory resulted in such a large literature investigating interactions among individual variables (see [1,6] for review) that the next direction in this experimental domain could be to relax some of the control restrictions to obtain additional insights into how people behave in more realistic settings and to use advantages that such non-laboratory experiments offer.

As a stepping stone between transferring from the highly controlled laboratory experiment to only loosely controlled field or natural experiments, it is necessary to investigate the replicability of the main effects in these experiments in the field and in laboratory settings [16]. For example, in [25] the author replicated in the field a standard experimental design used in the environmental policy experiments. He found contrasting results to the previous laboratory studies by [26,27]. Ref. [28] postulated that “one highly important question about the external validity of experiments is whether the same individuals act in experiments as they would in the field.” Authors in [29] investigated the differences in behaviour in computerised matrix games between student, professional card game players and professional football players, conducted in the laboratory and in the professionals’ natural environment. They found that both professionals and students fall prey to cognitive biases when in the laboratory. They surmised that professionals come to the laboratory with the pre-learned skills and knowledge and, when exposed to the same role as in real life, they transfer this knowledge to the laboratory task. In contrast, when exposed to a novel task or novel environment, the professionals fall prey to the same biases as students, indicating that the environment in which one preforms a task may have a crucial role on the performance.

Authors in [30,31,32] advocated the use of field studies for economic experimentation as opposed to laboratory experiments that according to the authors lack generalisability to the real life behaviour. Their work was heavily criticised by [33], who reviewed a number of studies that directly compared field studies with their laboratory counterparts. According to [33], by 2011, there were only 6 studies designed for direct field-lab comparison. None of these studies used experimental asset markets but there was a high correlation between the lab and field results.

## 3. Preliminary Considerations

### 3.1. Between the Laboratory and the Field

Borrowing from [16], we now discuss five factors that can be used to define the taxonomy of field versus laboratory studies.

First, in the laboratory, usually the participants are students, while field studies would seek to recruit participants within a particular target group. Second, in a field experiment, participants (e.g., finance professionals) can bring specific knowledge about trading, which could affect the experimental market. Third, in the laboratory, participants usually trade abstract assets, while many field studies and natural experiments (i.e., studies that collect naturally occurring data) may use naturally occurring goods. Fourth, in [16] they point out that the stakes in experimental asset markets are usually not comparable to the real traders’ payments. Fifth, the nature of the task defines whether an experiment is a field study or a laboratory experiment. For example, implementing the SSW design [34] in the field (i.e., on a trading floor) would result in an artificial task, even if conducted at a professional site instead of at the university, and would remain a kind of laboratory experiment.

Our laboratory–field comparison focuses on evaluating whether the strictly controlled experimental environment is necessary for obtaining reliable results. We test whether implementing the experimental task in the participants’ natural environment could potentially yield richer data on people’s behaviour concerning stock markets. For this purpose, we recruit the same type of participants (students with uniform educational background), who are in general naïve with respect to trading with no or little experience, in both the laboratory and field study. To equalise the level of information for the field and the laboratory participants, we descriptively present the information that the participant in the field study could experience over a longer period of time. This procedure emphasises the direct difference between experiencing a particular process rather than being presented with its description. This difference can influence people’s decision making [35]. However, due to time constraints, providing descriptive information about the task at hand is a standard procedure in laboratory experiments. Therefore, our study could potentially reveal the impact of the natural environment experienced over a long period of time on the market dynamics.

Further, in our study, students trade the same goods in both settings. The assets correspond to the lecture slides of the professor (see below). Therefore, for the participants in the field study, the assets should be similar to naturally occurring goods, while for the participants in the laboratory the securities are an abstract part of the story of the experiment. In both settings, our participants are rewarded competitively and appropriately to the environment in which they act—bonus grades that could help one pass a course (classroom-based field study) and monetary compensation that is substantially higher than student hourly wages (laboratory experiment). Students enrolled in the class may find it natural to receive grades for the task completed along their coursework, while laboratory participants should be used to receive money for performing tasks. In both settings, we strictly enforce the same payoff function.

One could argue that the framed field study we describe is nothing but a classroom experiment. However, the main goal of classroom experiments in economics is to demonstrate to the students the law of finance and economics for pedagogical purposes. Our goal is to test whether introducing an engaging, entertaining and partially educational task to student groups can result in valuable data that could be difficult to collect in the laboratory. In a second step, we adapt the field experiment conducted in a classroom to the controlled laboratory conditions, while preserving the goal and the procedure of the task but in very different conditions, field versus laboratory. Therefore, two groups of participants perform the same task. One group works in a controlled environment within a short time frame. The second group acts “in the wild” where the task can be performed at their time of convenience and with engagement in the trading environment.

### 3.2. Incentive Compatibility

An additional aim of the present study was to investigate whether different types of incentives proposed to participants to perform experimental tasks lead to compatible results. This topic has gained a lot of attention in experimental economics and resulted in a large literature (see [6,36] for reference). In economic thinking, the true behaviour can only be elicited if the appropriate monetary incentive is applied. However, Ref. [37] finds that different incentive structures can lead to the same results regarding belief elicitation. Authors in [38] claim that the intrinsic motivation of participants can be so high that incentives do not matter or even can be harmful for the task, resulting in over-learning and putting “too much effort”.

In order to resolve the debate between psychologists and economists about whether monetary rewards have positive (economic view) or negative (psychological view) impact on performance, Ref. [39] conducted a set of economic experiments in which they found non-monotonic relationship between monetary payment and performance. Their results indicate that high payments increase performance while small payments yielded poorer performance than no rewards. In [40] they demonstrated that the brain’s reaction to reward is context-sensitive and scales the reward with respect to the possible range of outcomes. Authors in [41] showed that higher hypothetical monetary rewards (i.e., the rewards presented as experimental money rather than small values of real money) result in higher activation of the brain regions responsible for processing rewards. In an fMRI-based study, Ref. [42] found that the same region of the brain—the medial orbitofrontal cortex (mOFC)—is activated when people receive tangible monetary rewards and when they imagine rewards that are important for them. Along the same lines, Ref. [43] showed that the same brain regions (i.e., the ventromedial prefrontal cortex) are involved in computation of monetary and social rewards.

These findings indicate that, on the neurobiological level, monetary or non-monetary rewards have to be well-suited to the context of the task and the scale of possible outcomes, while real tangible money is not necessary to elicit good performance in a task. Along these lines, authors in [20] criticise monetary compensation by not accounting for other motives, such as the need of performing well in the group. In their review on the neural underpinnings of intrinsic motivation, Ref. [44] proposed a new scientific direction—the neuroscience of intrinsic motivation—which highlights personality, biological and physiological differences in how individuals exhibit intrinsic motivation (i.e., motivated by one’s intrinsic motives such as curiosity) as opposed to extrinsic motivation (i.e., motivated by external stimuli such as money). This proposition is, in particular, motivated by the observations that intrinsic motivation tends to elicit performance in a more persistent way than extrinsic motivation.

Authors in [45] made a direct comparison of the effectiveness of monetary vs. credits in an individual investment and found that when compensated with credits, women obtain higher earnings than men, while there was no gender difference when participants are compensated with money. Ref. [46] focused on comparing monetary vs. credit rewards in laboratory-based experimental asset markets. They utilised the experimental design by [34], where participants in both conditions (cash vs. credits) completed the task in the laboratory. Ref. [46] concluded that the formation of bubbles in both conditions was the same, independently of the payment method. They point out that this finding allows for an important extension of this type of experiments to include larger numbers of participants because the lower budget required for experiments with credit points compensation schemes.

Another important aspect of incentives is the way the final compensation is computed. Ref. [47] recalled that experiments with multiple trials can implement a variety of payment by the experimenter to the participants: (i) payment based on a single randomly selected round, (ii) payment based on the cumulative performance over all rounds, (iii) payment of only a subset of selected participants or to all of them. Overall, their investigation shows that paying either for a subset of trials or to a subset of participants is the most effective to motivate participants to perform.

Here, we propose that the compensation scheme should be appropriate for a particular setting and group of participants to be compatible with their intrinsic motivation to perform in the task. According to [48], grades work like monetary rewards. In our study, 0.5 of a grade point is valuable and can be decisive of passing a course. The Swiss academic grading system has 6-point grades, with 6 being the maximum grade, 4 being passing grade and 1 being the lowest (see explanation of the Swiss grading system here: https://www.swissuniversities.ch/en/higher-education-area/swiss-education-system/grading-system/). In the laboratory experiment, we offer monetary payment that, for the best performing students, is over 1.5 times as much as a standard hourly payment for a student job (27 Swiss francs per hour, in year 2016). In this study, we can directly compare the behavioural effects in experiments that have the same compensation function with conversion to different assets (i.e., money vs. grades), such that each of these compensation schemes is compatible with the experimental setting at hand.

## 4. Method

### 4.1. Field Study

The field study described here corresponds to Experiment 2 in [17], which provides in depth details of this experiment. We decided to replicate Experiment 2 in the laboratory, because it included important improvements in comparison to Experiment 1.

In a trading experiment, students of the Financial Market Risks course in Fall semester 2015 in the Department of Management, Technology and Economics at ETH Zurich were trading the lecturer’s slides and had to predict the slide on which the professor will finish the next lecture. The professor always prepares more slides than he needs and he does not know precisely himself on which slide he will finish the lecture. The number of slides per lecture varied between 78 and 168. Each security on the market corresponded to three consecutive slides. For the purpose of the experiment, every week, the professor uploaded the slides to a student portal a week in advance. A total of 122 (55% of the enrolled students) students participated. Participation was voluntary and had no negative impact on the students’ final grade. At the end of the semester, the best 25% of the students received 0.5 bonus credit point, the second best quartile would receive 0.25 bonus credit point, while the worst half of the students would receive no bonus.

Each experiment had four experimental rounds, each round lasting 6 days (Tuesday–Sunday) preceding the class. The class would take place at 10:15 a.m.–12:00 p.m. on Monday. At the end of the lecture, the professor announced the ending slide. The security corresponding to this slide would pay out a dividend of 100 units of experimental currency, while all other securities would be priced at 0. Therefore, to perform well in the task, one would have to trade to either obtain a lot of cash and/or correctly predict the ending slide by buying as much as possible of the corresponding security.

The design is characterised by a few features that should mitigate mispricing: (1) equal endowment and a fixed deferred dividend, (2) small cash-to-asset ratio, (3) trading time lasting six full days, and (4) possibility to communicate among the players and an open order-book. Despite these features, Ref. [17] found substantial mispricing of the market. This mispricing pattern departs from the typical “bubble-crash-scenario” often found in the SSW experimental asset markets [34] but these differences could partially be attributed to the differences in dividend structure. In addition, the prices reflected the traders’ ex-ante belief about the success of each of the securities. The initial distribution of the price demonstrated a communal agreement about which securities are “good” and “bad” despite the Knightian [49] uncertainty and lack of fundamental value. Recall that Knightian uncertainty refers to a situation in which outcomes of events are known but probabilities of their occurrence are not known and/or cannot be computed.

### 4.2. Laboratory Experiment

#### 4.2.1. Participants

Thirty six students of a Swiss University were recruited over the UAST database (https://www.uast.uzh.ch) to participate in a trading competition experiment. From the UAST participant pool, we selected students with majors (engineering, natural sciences and social sciences such as management and economics) that matched the background of the participants in [17]. In the invitation e-mail, we informed participants that, in the study, they would compete against other participants and that the compensation will be competitive. The e-mail included information about the possible minimum and maximum payment. The point of providing this information was twofold: to obtain self-selection of participants in similar ways as it occurred in the field study and to comply with ethical guidelines of conducting behavioural experiments (i.e., informing participants about the purpose of the study). Seventeen (47%) of the participants were female, which reflects the standard recruitment procedure in laboratory experiments. The age range was 18 to 32 years (mean age = 24 years). The number of participants corresponded to the full capacity of the laboratory. None of the participants attended the course of Professor Sornette and all were unfamiliar with his lecturing style. This assured that all participants had the same base knowledge about the task, which is usually the case in laboratory experiments. The maximum capacity of the Decision Science Laboratory (cf. DeSciL: https://www.descil.ethz.ch) of ETH Zurich determined the number of participants.

#### 4.2.2. Procedure

Participants arrived at the laboratory and were promptly seated at 2pm to randomly assigned seats in the laboratory room. After reading the instructions (see Supplementary Materials) the participants watched a movie describing the professor’s lecturing style and video instructions on how to use the trading platform, both lasting about 15 min in total. Next, a trading task consisting of one practice round and three experimental rounds with the trading time of 10 min each followed. The practice round did not count to the final rank and participants were informed about that. In each round, participants received the endowment of 300 units of experimental currency and 3 units of each security available on the market which corresponded to a loan worth 600 units of experimental currency that had to be repaid after the round finished. After each trading round when the ending slide and the corresponding security were announced, the winning security was priced at 100 while other securities were priced at 0. There were 117, 168, 157 and 144 slides in the practice round and rounds 1–3 respectively, which corresponded to 39, 57, 54 and 49 securities (3 slides per security). The winning securities were 15, 15, 23 and 21.

The content of the slides provided some information about the topics covered in the lecture. However, according to our qualitative and quantitative analysis of the professor’s slides that we describe in Appendix A.1 and Appendix A.2, there were no features that would help to predict with 100% accuracy when the professor would finish the lecture.

Before and after trading in every round, the participants were asked to submit their belief about the success of each slide, using the roulette belief elicitation method [50,51]. To submit their belief, participants were asked to allocate 100% of their belief among all available securities, in any fashion that they wanted, as long as the sum of the allocated beliefs summed to 100. For that purpose, they were presented with a bar graph with all securities listed on an x-axis with uniformly assigned weights to each security. The participants could freely adapt these weights according to their true beliefs. After the trading task, the participants completed a short questionnaire including demographics, trading strategies and the illusion of control [52].

**Figure 1:**
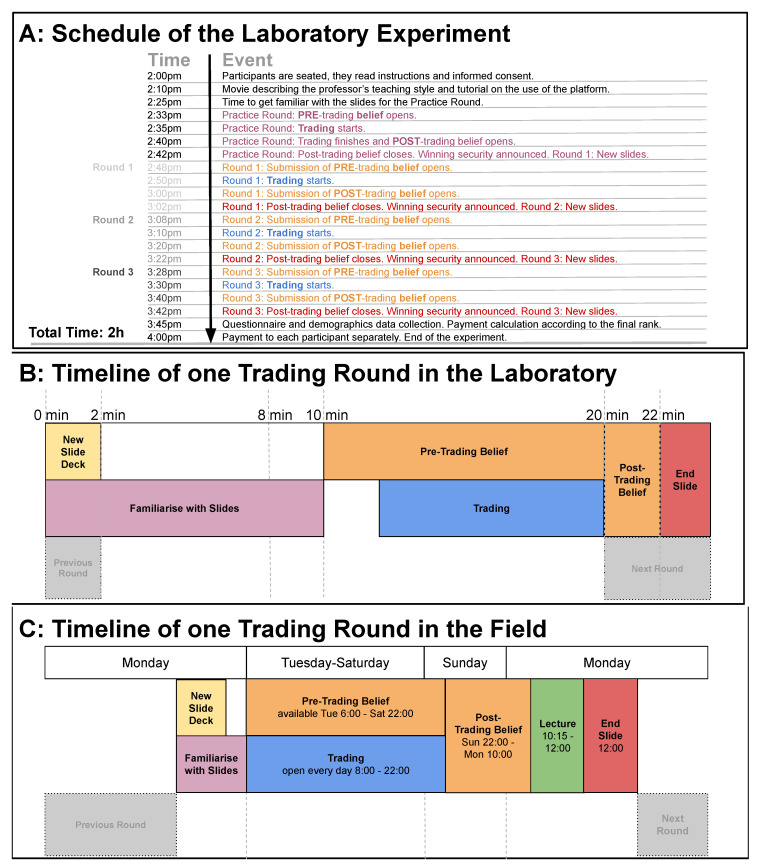
(**A**) The detailed schedule of the laboratory experiment including the exact timing that was the same for all participants; (**B**) Timeline of one trading round of the laboratory experiment; (**C**) Timeline of one trading round of the field experiment. The elements of the timeline are colour-coded.

The experiment followed a fixed time schedule that had to be obeyed by all participants. The exact timing of the schedule is provided in Figure 1. Each of the steps of the schedule were announced to the participants in writing on a black screen of their computer. We presented the information to all participants at the same time. Participants had access to the previous rounds and their account balance at any time during the trading task. During the experiment, the participants were allowed to take notes on a blank sheet of paper. The notes were collected by experimenters and were anonymous such that they were not assigned either to the real name or the experimental ID of any person attending the experiment. The scanned notes can be downloaded from https://osf.io/6jm9p/.

Before conducting the main experiment, we conducted three pilot studies with 6–12 student traders in the room. We do not report the results of these pilot studies because markets in these studies were not liquid enough with such a low number of participants. The purpose of the pilot studies was to set technical issues of the experiment, such as timing. During these pilot experiments, we calibrated the length of the individual trading rounds and the length of the whole experiment so that the whole experiment took not longer than two hours. The experimental procedure has been approved by the ETH Zurich Ethics Committee.

#### 4.2.3. Compensation

As in [17], for each round, the trading platform provided a ranking. The market was reset after every trading round and no assets were carried over to the consecutive round. The final rank was calculated based on the sum of earnings in each of the three trading rounds.

The best 25% (thus 9) of the participants in the final rank received a bonus of 60 Swiss francs (worth approximately 60 U.S. dollars), the second best 9 participants received a bonus of 30 Swiss francs and the worst 18 participants did not receive any bonus. This bonus scheme was intended to correspond to the payment of 0.5 and 0.25 of the grade credit points awarded in the field study as described above. All participants received a show-up fee of 30 Swiss francs, which was compliant with the rules of the Decision Science Laboratory of ETH Zurich. Therefore, the top performing students received 90 Swiss francs for a 2-hour experiment.

## 5. Presentation of the Main Results

For the purpose of direct comparison of the laboratory and field studies, we provide results from the laboratory and contrast them with the findings from the field study presented in [17]. Each comparison comes with a discussion about the similarities and differences between the two experimental settings. Please note that, while we expected differences between the laboratory and field settings, we did not have clear expectations on the nature of these differences because our investigation provides the first such direct comparison for a study with a large number of participants and of traded securities. We further summarise this analysis in Section 6. Data from the field and the laboratory experiments are published on the Open Science Framework at https://osf.io/6jm9p/.

### 5.1. Comparison and Quantification Criteria for Assessing Similarities and Differences between Experimental Environments

We test for the existence of the same stylised facts in the laboratory and in the field experimental settings. Also, we investigate the differences in the processes and dynamics of how these stylised facts appear in the laboratory and in the field. Due to the fact that the two experiments differed in duration and number of participants, hypothesis testing using neither the frequentist nor Bayesian approach is conceptually feasible to investigate similarities and differences between the laboratory and field environments. Hence, we use Jensen-Shannon divergence (cf. JSD) to measure information similarity between two distributions, such as price and belief distributions in the laboratory and in the field settings. JSD is a symmetric and bounded metric of the difference between the mixture entropy and the entropy of the mixture of distributions [53]. Therefore, JSD of the price distribution in the market reflects the entropy of the market. For two probability distributions *P* and *Q*, Jensen-Shannon divergence equals:

(1) $$JSD\left(P,Q\right)=H\left(\frac{P+Q}{2}\right)-\frac{H(P)+H(Q)}{2},$$

and H(X) is the Shannon Entropy of a probability distribution *X* with density $x_{i}$ for assets i=1,2,…,n, denoted as:

(2) $$H\left(X\right)=-\Sigma_{i=1}^{n}x_{i}\log_{2}x_{i}.$$

Another way to describe disorganisation of the market is to measure its mi-pricing. To quantify market mispricing, we use the Relative Deviation (cf. RD) [54], which in the experimental asset markets is used as a “bubble measure”. RD measures raw price deviations from the rational market price. RD indicates whether a market was over or under-valued such that RD=0 implies no mispricing and the larger the deviation from 0, the larger the mispricing is. To analyse the pricing rationality of the market, we calculated three market indices: index 1—sum of security prices in the market, index 2—sum of highest bid offers and index 3—sum of lowest ask offers. Due to the fact that the dividend pays 100 units of currency, index 1 should not exceed the value of 100 and for the market to be rationally priced, index 1 should equal to 100. If index 2 exceeds 100, or index 3 is lower than 100, there would be a straightforward arbitrage opportunity against positive and negative bubble on the market respectively. Therefore, for *N* periods *p*, we calculate RD of index *i* (i=1,2,3) according to the formula:

(3) $$RD_{i}=\frac{1}{N}\sum_{p=1}\left(index_{p}^{i}-100\right)/100.$$

### 5.2. Trading Activity

**Figure 2:**
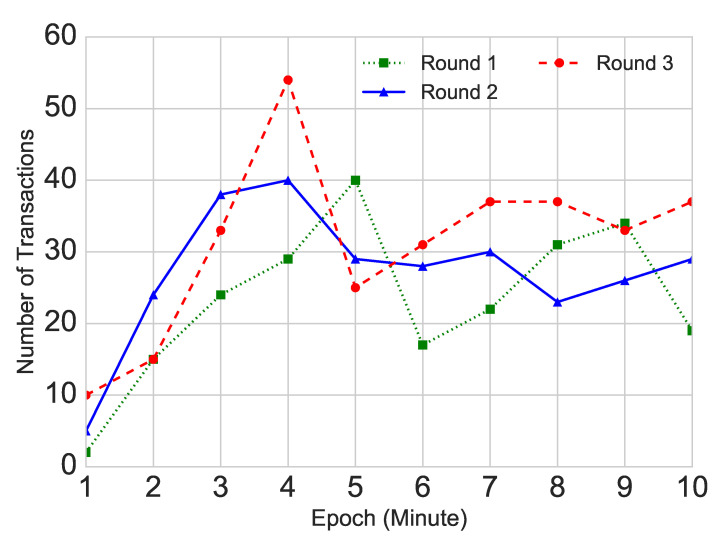
Number of transactions per minute in three rounds. The figure shows how the number of transactions changes during the trading time.

We observe an increase of participants’ activity from the first to the third round. The total number of orders increased from 676 in Round 1, through 844 to 922 in the final trading round. The average trading volume of each security is 6, 9.8, and 12.7 shares for round 1–3 respectively, which are 91%, 81%, 69% lower than the field experiment. As shown in Figure 2, the number of transactions in the laboratory increased within the first 1–5 min (5 min equals half of the trading time), when it reached the peak and then fluctuated at around 30 transactions per minute. This indicates that participants learned the task and started to react quicker in later rounds. This pattern of trading activity in the laboratory is in contrast to the trading activity in the field experiment, where the number of orders in each round decreased across rounds and the activity within each trading round had a clear cyclical pattern, with the daily peaks of activity in the morning and in the evening and the weekly peaks of activity just after the market opened and just before it closed (similarly to real financial markets). We do not observe such patterns in the laboratory.

On average, each student submitted 18.8, 23.4 and 25.6 orders in rounds 1–3. This indicates very high activity during the short trading periods lasting 10 min, compared to the classroom setting with the average number of orders per students within the 6-day period would equal 34.1, 26.5 and 19.7. We surmise that this increase in activity in the laboratory was related to learning and improving at the task. In contrast, the decreasing activity in the field could have resulted from the lack of interest in the task or improvement of trading strategies such that one would become more efficient with fewer trades. To correctly disentangle these effects, we conducted a follow-up experiment in Fall 2016 described in [17], where we found no difference in trading activity of experienced student traders during trading rounds lasting six days and two hours.

**Figure 3:**
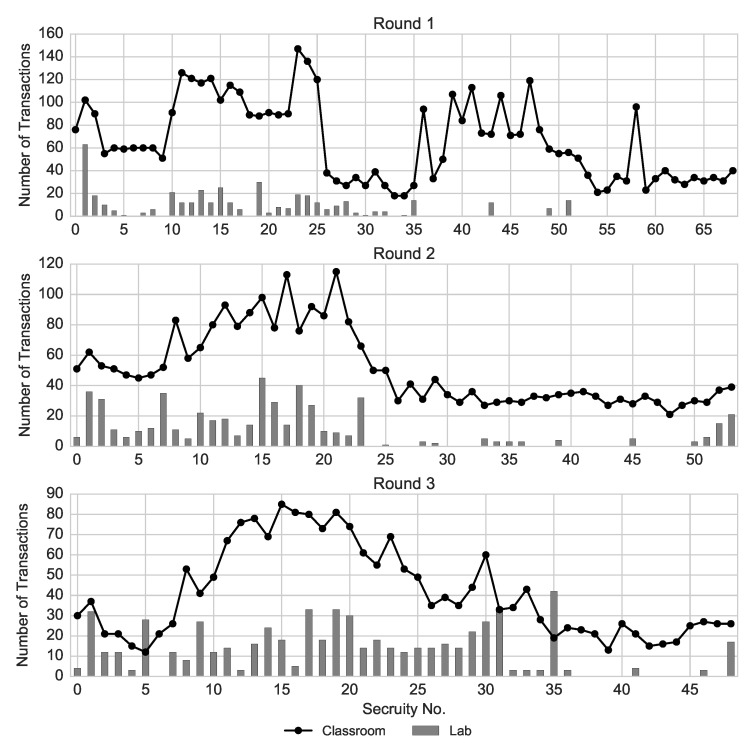
Trading volume of each security on the market in the laboratory (bars) and in the field experiment (line).

Figure 3 shows the trading volume of each security in the laboratory and in the field experiment. Similarly to the classroom setting, the prices in the laboratory market were strongly correlated with the trading volume (r=0.73,0.81 and 0.91, p<0.001 for Rounds 1–3). While, in both settings, the security listed as the first one (i.e., left-most) has a relatively high volume, in the laboratory the volume of that security was higher relative to the volume of other securities. This is especially pronounced in Round 1, where the first security on the list (i.e., Security 1) was traded twice as much as the next most traded security. In addition, in all three rounds, securities with larger numbers (on the right tail of the probability distribution) exhibit little or no activity. This is due to the limited time of laboratory trading rounds that restricted exploration and exploitation of all available securities. Additionally, in Appendix B, we provide a summary of the self-reported measures describing trading activity and strategies.

### 5.3. Market Prices and Participants’ Beliefs

**Figure 4:**
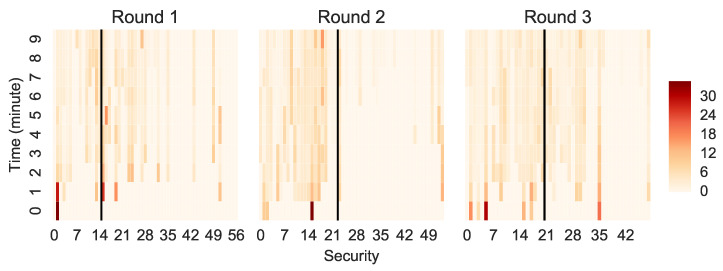
The evolution of security prices over time for the three trading rounds. The price distribution emerges within the first 3 min.

Figure 4 shows that the price distribution emerged in the first 30% of the total trading time, compared to the 6% of the available trading time in the field experiment. However, in absolute terms, the price emergence in the laboratory was very quick as it took only 3 min, likely forced by the fact that all participants had a strictly designated limited trading time.

Further, in Round 1, only Security 1 had a price in the first minute of the trading round. In addition, the securities that were priced early during the trading were much more expensive than the securities for which the price is established later in the trading round. The prices of these first securities diminished after minute 3 of the trading. We did not observe a similar pattern in the field experiment. In the laboratory experiment, 23, 17 and 10 (40%, 31% and 20% of available securities) securities remained without price in Rounds 1–3, in comparison to none in the field experiment. The fact that fewer securities remained without price across rounds shows that participants learned to explore all available securities and traded them.

Based on the median split of the final price at the end of a trading round, we distinguish between the “expensive” (i.e., good and possibly paying out the dividend) and “cheap” (i.e., bad and possibly not paying out the dividend) securities. Once the prices of the securities were established, the “expensive” securities remained expensive and the “cheap” securities remained cheap till the end of each trading round, which replicates the effect observed in the field experiment. This conclusion is confirmed by the JSD measuring divergences between the distributions of two consecutive minutes (see Table 1) ranging between 0 and 1, such that values close to 0 indicate almost identical distributions and values close to 1 indicate substantially different distributions. Thus, the JSD also confirms that the price emerges quickly, as the JSD falls below 0.1 after 5, 4, and 3 min for rounds 1–3 respectively.

Table 2 demonstrates the entropy values of the pre- and post-trading belief distributions and of the market distributions in the laboratory and in the field settings, as well as the JSD between the laboratory and the field. According to the table, the entropy of market price distribution in the laboratory is always larger than those in the field, while the entropy of belief distributions is always smaller than those in the field setting. This implies that the market distributions in the laboratory are less concentrated than the those in the field experiment. According to the JSD between the distributions of the laboratory, belief distributions in the laboratory are similar to those in the field (i.e., JSD<0.1), showing that traders in the laboratory and in the field experiments have similar beliefs about the final results. However, the JSD between the market distributions in the laboratory and the field experiments are much larger, providing another evidence that the traders’ beliefs have not yet been fully reflected in the laboratory market distributions, possibly due to the much shorter trading time than in the field.

**Figure 5:**
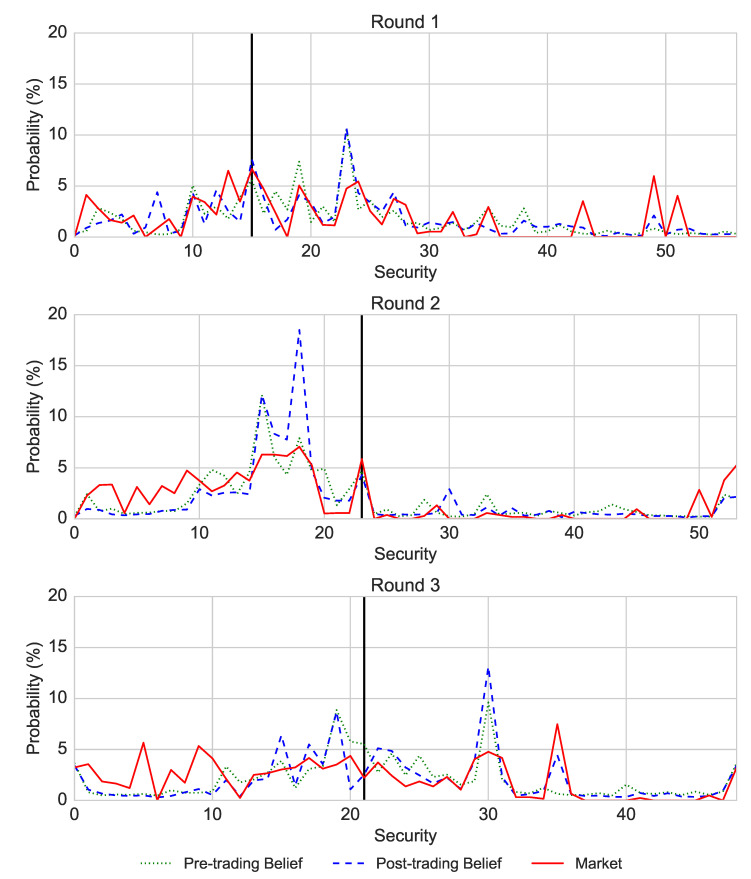
Distribution of securities’ prices, pre-trading and post-trading beliefs across 10 1-min epochs. For each epoch, we calculated the median price for each security. These median prices were then averaged across all 10 epochs. These prices were normalised so that their sum is 100. The pre- and post-trading beliefs were obtained by averaging the submitted beliefs across all 36 participants.

This price emergence resulted from the aggregated initial beliefs of the market participants. According to Figure 5, the average pre- and post-trading beliefs were very strongly aligned with the price distribution in each week. The post-trading distribution was more strongly correlated with the price distribution than the pre-trading belief (Pearson correlations of the price with the post-trading belief: r=0.62,0.71,0.51; Pearson correlations of the price with the pre-trading belief: r=0.56,0.71,0.38, p<0.001 for all correlations), while the beliefs were more correlated with each other than with the price (r=0.79,0.83,0.83, p<0.001 for all correlations). This replicates the corresponding finding from the field experiment. As outlined in Equation (4), for each round, we implemented a regression analysis demonstrating that the difference between the post-trading belief and the market can be predicted by the difference between the pre-trading belief and the market:

(4) $$Belief_{post-trading}-Price=\beta_{0}+\beta_{1}\times \left(Belief_{pre-trading}-Price\right)$$

In all three rounds $\beta_{1}$ ($\beta_{1}$ equalled 0.70, 0.84, 0.80 in Rounds 1–3) was significant at p<0.001 and the percentage of explained variance was medium and high ($R^{2}$: 0.57, 0.38, 0.64). The dependent and independent variables in this regression are expressed as differences between beliefs and the marked distributions to avoid the multicollinearity problem.

Further, the peaks of the distribution for each week were always the lowest for the price distribution, second highest for the pre-trading belief and the highest for the post-trading distribution. This is in contrast to the field experiment, where the peak of the price distribution was always higher than the peaks of the belief distributions. This means that, in the laboratory, the beliefs of the market players were directed towards particular securities more than the market (showing a coordinated opinion of the players), while it is the opposite in the field experiment.

**Figure 6:**
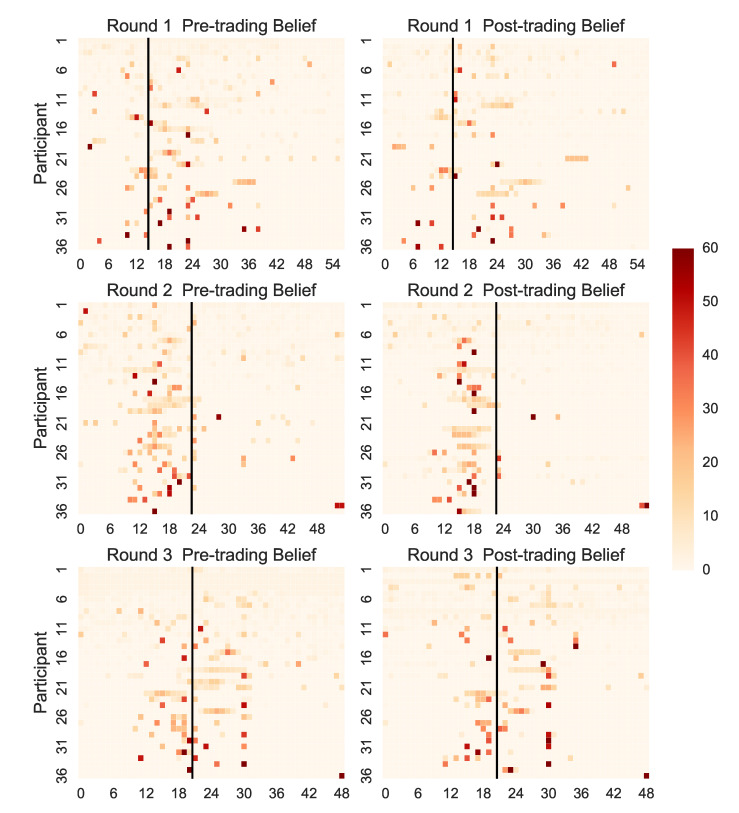
Heat maps of pre- (**left**) and post-trading (**right**) beliefs, such that each cell corresponds to the belief assigned by one participant to one security. The individual belief distributions are sorted in a decreasing fashion, according to the number of securities with non-zero weights.

Figure 6 shows that the beliefs of individual participants were convergent on which of the securities would pay out a dividend. The securities that were assigned with more weight are close to the realised securities. Overall, the beliefs in Round 1 were more dispersed than beliefs in Rounds 2–3 and most of the belief were assigned close to the executed securities. This finding is consistent for the two experimental settings.

### 5.4. Mispricing and Market Rationality

**Figure 7:**
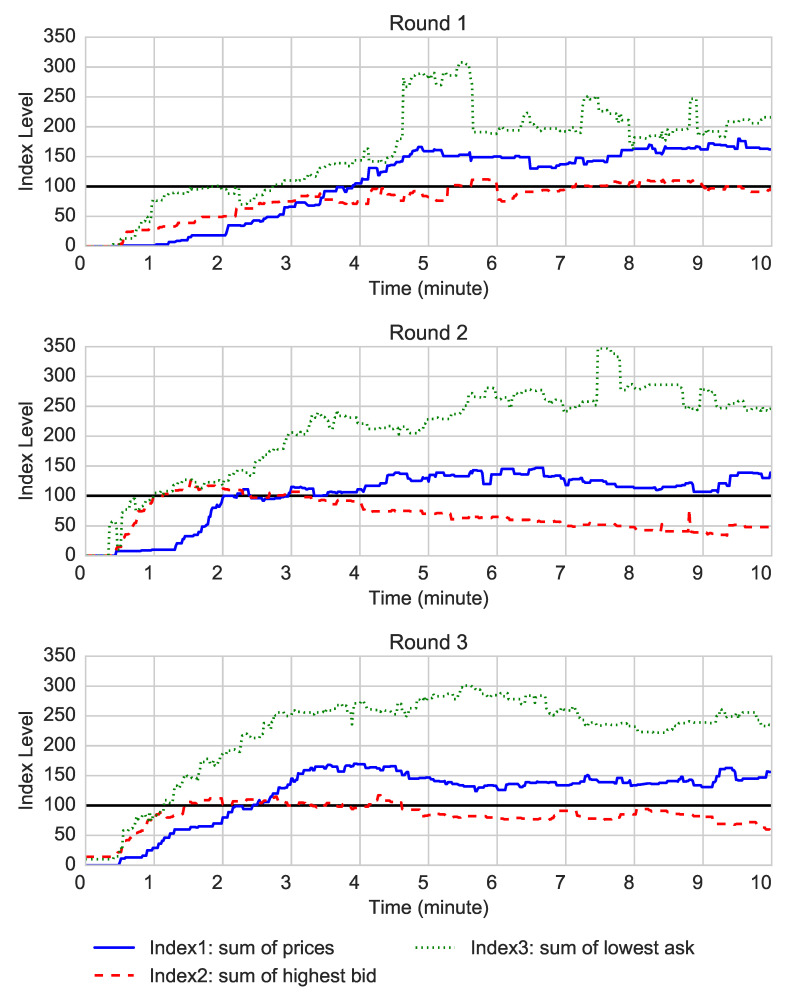
Evolution of three market indices corresponding to the sum of all securities’ prices, sum of the highest bid prices, and sum of the lower ask prices. The sum of all security prices should be 100 but there are several pronounced deviations from this normative prediction.

Figure 7 shows the evolution of the three indices across 10 min of each trading round. First, the market was overpriced in all three experimental rounds, which is confirmed by the RD presented in Table 3. The negative RD of index 2 in all three rounds are due to the fact that many securities remained without price, where the number of priceless securities was larger in the laboratory than in the field. This could potentially be explained by the much snorter trading time in the laboratory. The bubble levels in the laboratory and the field experiments are similar. However, this overpricing was not as pronounced as in the field experiment. In Round 1, index 1 exceeded 100 only after 4 min of trading (i.e., 40% of the trading time) and stayed at the level of about 150. In Rounds 2 and 3, index 1 exceeded 100 after about 2 min. In the laboratory setting, we did not observe decrease of this mispricing across rounds. As the average bid prices were smaller than 100 almost all the time, there were no obvious arbitrage opportunities in the laboratory setting *on average*. However, given that the prices were at times very large for some securities, in the self-reported questionnaire, seven participants reported that they applied an arbitrage strategy, selling the securities with high prices. In the field experiment, we observed one strong arbitrage opportunity in Round 1 and one in Round 4, in the sense that the best bid price became transiently larger than the best ask price. The development of all three indices is very similar in all three trading rounds.

Second, the over-pricing was particularly well characterised by the time intervals during which index 2 becomes larger than 100: in Round 1 briefly at the end of the sixth minute and during the eight and ninth minutes, in Round 2 during the second, third and fourth minutes, and in Round 3 from the second to the fifth minute. The fact that the best bid was larger than 100 means that any transaction had to be concluded at a price that would result in an aggregate price significantly above 100, in clear violation of the rationality and fair value argument.

In the field experiment, the overpricing was the highest during the first half of the day when the market opened (i.e., 5% of the trading time) and it decreased towards the end of each trading round. In addition, the mispricing diminished across rounds. We attribute these differences to the time constraint and late formation of the price distribution in the laboratory.

### 5.5. Trading Performance and the Illusion of Control

In the final questionnaire that followed the trading task, 18 participants (50%) responded that they realised that the market index should equal 100. Three of the seven persons that reported implementing arbitrage strategy were in the top quartile, two were in the second best quartile and only the remaining two did not receive any bonus, but were in the third quartile. This supports the observation that there were some arbitrage opportunities only based on recognising that the market (and a number of securities) were overpriced.

In the post-trading questionnaire, one participant reported to have had a few years of experience in trading, two people reported having 3–6 months experience (an equivalent of an internship) with trading, while others had no experience. The person with a few years of experience was fifth on the final rank.

In contrast to the field setting, we found no correlation between the number of submitted orders and participants’ earnings. There was only one person (an outlier), who not only submitted substantially more orders ($N_{orders}=156$) than other participants (Range: 16–119, M=68), but also, this person had a substantially higher total earnings (Earnings=3529) than the rest of the participants (Range: 2471–1041, M=1800). Therefore, this participant had rank 1. This suggests that the laboratory setup promotes more of a gambling atmosphere with insufficient time to ponder and evaluate the options as well as keep or recover a cool trading mind.

Further, for each participant, we calculated the primary illusion of control [52], which relates to the belief that one has a control over the outcome of the stochastic process, the secondary illusion of control, which defines that a person aligns themselves with having extraordinary skills such as “feeling lucky moments”. For each participant, we computed the average of responses from the questions corresponding to each subscale (primary and secondary), where each question was measured on the scale 1–10. The total score of the illusion of control is the average from all questions in the survey. Overall, all participants had a low primary (M=3, Range: 0.5–5.67) and secondary (M=1.44, Range: 0–5.33) illusion of control, as well as the total score (M=2.58, Range: 0.8–4.8) of the illusion of control. The last question of the illusion of control questionnaire asks on a scale 1–10 whether “It was all chance”. Six people replied 1 on this question meaning that they believed that their performance was completely attributed to their actions. Only three people responded 10 (maximum value) indicating that they believed that they had no influence on their performance. The distribution of responses was slightly positively skewed, with the median of 4 and mean equal 4.06.

We found a moderate correlation between the final earnings at the end of the three trading rounds and the total illusion of control (r=0.44,p<0.01). This correlation was driven by the strong correlation between the secondary illusion of control and the final earnings (r=0.57,p<0.001), while there was no correlation of the final earnings and the primary illusion of control. Given the fact that the survey of the illusion of control was preceded by the trading task and that the participants generally had no trading experience, we interpret that those participants, who received better scores in the trading task, attributed their success to their skills such as “feeling the market”. This relation was also reflected in the negative correlation between the final rank and the total illusion of control (r=−0.57,p<0.001), the negative correlation between the final rank and the secondary illusion of control (r=−0.71,p<0.001) and no correlation between the final rank and the primary illusion of control. There was no correlation between the trading volume or number of orders and any measure of the illusion of control, which means that the illusion of performing well was attributed only to the final results of the trading.

Ref. [52] found that higher illusion of control was correlated with people’s prior beliefs about the outcome of a gambling task that their participants performed. In our experiment, we find that participants in the laboratory condensed their beliefs to fewer securities than the participants in the field experiment. The distribution of the prior beliefs had a larger peak and thinner tails than in the field. We surmise that the laboratory participants formed more extreme beliefs while being less confident about these beliefs and their actions.

## 6. Discussion

### 6.1. Common Findings in the Laboratory and Field Experiments

In this study, we adapted a complex field experiment involving an experimental asset market to laboratory conditions. We replicated the procedure of the field experiment in the highly controlled experimental setting for the purpose of testing the relation between the laboratory results and complex trading environment.

In the laboratory experiment, we replicated a number of key effects found in the field experiment. First, we observe that the initial price emerges early during the trading round and the price distribution stays relatively constant until the end of the trading time. Second, this price emergence is a result of the initial belief of the market participants. The post-trading belief was more correlated with the price distribution of the securities than the pre-trading beliefs, but the two beliefs correlated more strongly with each other than with the price distribution. Third, we observe significant mispricing, despite the fact that half of the participants realised that the market was overpriced.

The fact that we replicated these behavioural effects in a highly controlled setting with time constraints highlights the robustness of the findings. This speaks in favour of the reliability of these results independently of the environment, in which the experiment was conducted. The most robust effect found across many studies is the market mispricing. It is worth noting that, in our laboratory study, despite the fact that 20–40% of the securities were not priced, the market was overpriced over half of the trading time. Surprisingly, the mispricing in the laboratory occurred at a relatively later point during trading (in percentage of total trading time) than in the field experiment, which at prima facie seems to contradict the Active Market Hypothesis [55] but may also be associated with the incompressible time for participants to make up their mind within the few minutes available in the laboratory.

In addition, we showed that, in the laboratory, the market forms even when the securities are abstract and the participants have minimum knowledge about the traded assets. Our participants formed an opinion (i.e., belief) about a stochastic process, with minimum prior knowledge about it. This questions the validity of the experimental findings, because the laboratory participants formed a more extreme opinion about securities and they had less knowledge about the underlying securities than the field participants who indicated more uncertainty in their beliefs. Previous psychological literature on long-term memory indicates that context and previous experiences can have a strong effect on judgement formation by accentuating some information [56]. Therefore, some contexts may potentially evoke stronger opinion formation. Experimental asset markets that do not involve forecasting (i.e., the SSW-like experiments) have much smaller context effects, which should make them easier to replicate. The fact that we observed mispricing in our laboratory and field experiments that had a rich context confirms the strength of the persistent mispricing across various markets and setups.

Despite the differences in market dynamics, the robustness of the main effects between the laboratory and the field study demonstrates that it is possible to relax some of the controlled measures in the laboratory in favor of additional advantages of field experimentation. For example, in the field study, there was no limit in the number of participants, while in the laboratory, we were restricted by the capacity of the laboratory. Additionally, allowing participants to complete the task from any place that they find convenient offers the possibility to record their behaviour in their “natural” environment and to run the study for a much longer time (i.e., four weeks instead of two hours). Thanks to the larger number of participants, the market in the field study was more liquid, which demonstrates that increasing the complexity of the task may require increasing the number of participants in each particular round and extending the duration of one round.

Our results confirm the hypothesis that the participation in an economic experiment should be endowed with the compensation scheme that is relevant for the particular experimental setting. We obtained the same key effects when compensating students enrolled in a class with bonus grade points and endowing laboratory participants with competitive amount of money. Our work extends the findings of [46] by demonstrating that credit-point compensation can be implemented not only in cumulative payments from the whole experiment, but also in a rank-based compensation. In addition, we show that this extension holds for experimental asset markets different than the classical experimental design by [34].

In this study, we purposefully used the same experimental materials (i.e., the professor’s slides) as in the field experiment in order to directly compare the two settings. However, this design allows for several extensions. For example, in order to investigate how important is familiarity with a particular stock, one could conduct an experiment in which students trade abstract stocks such that the one paying out the dividend would be chosen according to a stochastic process. Another extension could test the predictive power of the market by asking students to predict a real life event such as outcomes of sports events. Imagine that each security corresponds to one athlete in the 400-meter run competition at the Olympics. Experiment participants could trade these securities before the run. In [17], we provide a complete overview of all variations of the initial experimental design that we implemented.

### 6.2. Observed Differences between the Laboratory and Field Setting

Despite replicating the main findings from the field experiment, we observed a few differences between the field experiment and the laboratory experiment. First, we did not replicate the effect of the decrease of mispricing across trading rounds. We surmise that this was due to the short trading time in the laboratory setting. In addition, there are substantial differences in the market dynamics between the laboratory and the field setting.

Second, the price distribution became stable at a later stage during the trading round in the laboratory compared to the field. It is important to note that the definition of “late” is a relative concept, measured as percentage of the total available trading time. In absolute terms, exceeding the rational price level after 2–4 min after the market opens is comparable to the time needed for bubble development in the experiments using the design by [6,34,36].

Third, many securities remained without price, which is not the case in the field setting. In addition, the price distribution is less “smooth” in the laboratory, which makes it difficult to judge the predictive power of the market. The differences in the price distribution are related to the short trading time and complexity of the task. Our results demonstrate that the time allowed for trading is a very important component that not only makes the market more liquid, but also gives the market players more opportunity to explore the complexity of the market.

Fourth, in the field experiment, we observed a characteristic daily and weekly cycles of trading activity. These fluctuations show that the market liquidity differs at different time points. For example, some orders were executed immediately when many traders were logged to the trading platform, while other orders had a longer waiting time or could be cancelled by the issuer, at times when few traders were active. In that logic, there were times at which participants could “think twice” and times at which they had to react fast. In the laboratory, it was impossible for the participants to thoroughly think about their strategies and they had to react fast at all times. This was reflected by more diversified self-reported trading strategies and higher trading volume of the first security listed in the platform.

Further, while transferring the field experimental design to the laboratory, we experienced a few challenges. First, given the rather large number of traded securities, the market was not as liquid as in the field setting, despite the high trading activity and our use of the maximum capacity of the laboratory. This points to the limitations of the laboratory experiments—implementing a large number of securities requires a large number of participants as well as long trading rounds. Implementing an online experiment would provide a solution to this problem. However, the experimenters would not be able to control what the participants really do. We propose that this high degree of control of the experimental setting introduces artificiality. In real life, traders constantly face distractions, check e-mail, browse the Internet, and are continuously subjected to a flow of news through various channels. Forcing participants to focus on one task only does not resemble the real markets. In contrast, the field experiment captures well this condition.

Next, using a realistic, complex trading platform requires teaching the participants on how to use it. The trading platform that we used in both experiments is a multi-tab software, which mimics some functionality of professional trading software. In order to make it possible for our naïve participants to use it, we created a video with instructions that worked as a 7-min crash course to the software. We cannot eliminate the possibility that some participants underperformed because they had to learn how to use the software “on the go”. This is another demonstration that a realistic trading task may be too challenging for a short laboratory experiment. Participants need time to learn how to use the software and how to perform well in the task [57]. We describe the xYotta trading platform used for this experiment in [17].

### 6.3. Motivation for the Changes between the Field and Laboratory Setting

In order to adapt the field study to the laboratory conditions, we had to make a few changes to the design. First, the main change was the number of participants reduced from over 100 to exactly 36. On the one hand, the laboratory setting allows for the control of an exact number of participants (In the field setting, the number of participants fluctuated across experimental rounds). On the other hand, the number of participants was strictly limited by the laboratory capacity, which in settings with low market liquidity caused by a large number of securities can pose an important problem. Indeed, our market had lower liquidity in the laboratory than in the field. In that sense, the field experiment has the advantage of measuring price emergence and development of complex markets with multiple securities. In addition, in real life markets, the number of traders is not controlled. Despite the standard criticism of non-laboratory experiment in which the experimenter “cannot control what participants are really doing”, the less controlled setting can shed more light on how people really behave.

Second, in order to make the participants learn to use the trading software with several tabs and to explain the relatively complex task for a short experiment, we had to present a manual on how to use the software in a form of a concise and comprehensible movie, while in the field setting we were able to provide a presentation of the software in the classroom. This is a general limitation of implementing realistic complex tasks in short laboratory experiments with participants that are not familiar with the task and software.

Third, due to time restrictions of 2 h that was partially dictated by the Decision Science Laboratory, all information had to be presented in a very coherent way and we had to reduce the number of trading rounds from 4 to 3. While this change reduced the number of obtained data and statistical power, the main effects held.

Fourth, in the laboratory, we presented a movie summarising the professor’s lecturing style while participants in the field setting could experience first-hand the professor lecturing. This change raises two types of criticism. The first arises from the description-experience gap [35], which states that people tend to under-sample the outcomes of events and make their decisions accordingly. In a similar fashion, it is likely that each participant experienced the professor’s teaching style differently, which could have impacted their trading strategies. In the laboratory setting, all participants received the same information about the professor’s teaching style. On the one hand, presenting the same information gives more control over the flow of the experiment. On the other hand, presenting information descriptively results in a standard criticism of artificiality of laboratory experiments in all behavioural sciences.

## 7. Conclusions

Does the behaviour of participants depend on the way entropy is induced in experimental asset markets? Despite the replication of the key results, we found the existence of substantial differences in the market dynamics in the two experimental settings. The key reason for these differences was the time constraint that limited the learning to trade and to use the software by the participants in the laboratory. In spite of this time limitation, and in the presence of an intrinsic uncertainty about the market fundamentals and very limited knowledge of the market process and of the securities, we observed a very high market activity and a rapid price formation dynamics in the laboratory conditions. These findings demonstrate differences in psychological entropy depending on the experimental context. Our findings pose the question of what information can reliably be extracted from trading experiments in the laboratory, where this is the only task performed by the participants in very unrealistic conditions.

The confirmation that the key effects of the field experiments were reproduced by the laboratory version, together with the fact that the field conditions did not suffer from the many unrealistic constraints, while presenting other findings better in accord with empirical observations in the real world, suggests that these new class of field experiments, as introduced by [17], can have a promising future. Nevertheless, the goal of this paper has been to raise researchers’ awareness to the fact that standard laboratory experiments may not mimic the behaviour of real complex financial systems. Alternative setups can be developed with intermediate levels of control and complexity that may help close the gap between the maximally controlled laboratory conditions and the complexity of the real financial markets. 

## Figures and Tables

**Table 1:** Jansen–Shannon Divergence of end of each minute in the laboratory study. For minute 1, the values correspond to the divergence between the price distribution and a uniform distribution.

	Minute									
Round	1	2	3	4	5	6	7	8	9	10
1	0.89	0.61	0.28	0.23	0.08	0.11	0.12	0.09	0.07	0.08
2	0.86	0.60	0.13	0.04	0.15	0.09	0.10	0.06	0.11	0.03
3	0.73	0.34	0.29	0.08	0.02	0.06	0.03	0.04	0.07	0.04

**Table 2:** Entropy (*S*) of pre- and post-trading beliefs and of the market distributions in the three consecutive round of the laboratory and field experiment and the Jansen–Shannon Divergence comparing the laboratory and the field experiment ($JSD_{LF}$).

	Round 1			Round 2			Round 3		
	S Lab	S Field	${\mathrm{JSD}}_{\mathrm{LF}}$	S Lab	S Field	${\mathrm{JSD}}_{\mathrm{LF}}$	S Lab	S Field	${\mathrm{JSD}}_{\mathrm{LF}}$
Pre-trading Belief	3.59	3.76	0.13	3.44	3.46	0.08	3.51	3.55	0.06
Post-trading Belief	3.59	3.62	0.17	3.18	3.25	0.07	3.38	3.51	0.08
Market	3.34	3.16	0.36	3.29	2.78	0.3	3.43	3.11	0.27

**Table 3:** Relative Deviation (RD) for the three market indices: the sum of prices (index 1), the sum of highest bid prices (index 2) and the sum of lowest ask prices (index 3) in the three experimental rounds in the laboratory and field experiments.

	Round 1		Round 2		Round 3	
RD	Lab	Field	Lab	Field	Lab	Field
Index 1	45%	30%	19%	42%	30%	27%
Index 2	−7%	−50%	−51%	28%	−25%	6%
Index 3	101%	178%	150%	87%	139%	82%

## Supplementary Materials

The following are available online at www.mdpi.com/xxx, Instructions to the laboratory experiment: “Trading Competition (2016)”.

## Appendix A. Details of the Experimental Method

### Appendix A.1. Materials and Apparatus

The movie describing the professor’s lecturing style was based on the two lectures (Lectures 1 and 2) professionally recorded by the university services in Fall 2015 (Experiment 2 of [17]). The movie included the most characteristic features of Prof. Sornette’s lecturing style and a written summary of these features. The movie can be viewed under this link: https://osf.io/6jm9p/. The features of the professor’s lecturing style, as displayed in the movie, are listed in the Supplementary Materials.

The selection of the features was based on the notes and observations of two Teaching Assistants (cf. TAs) that were present during the professor’s lectures. Based on the notes systematically taken by one of the TAs, the number of slides, time spent per slide and time spent for each topic were not related to how many slides the professor would cover. The TA’s notes and classification of the slides into topical chapters were used for qualitative and quantitative analysis of the slide content. This qualitative and quantitative analysis supported the hypothesis that the lecturing style of the professor is a truly stochastic process with a number of characteristic features. The content of the slides represents the topics covered in the lecture by the professor, but the actual message of each slide is difficult to judge before seeing the professor present the slides in the lecture.

To define securities in the market, we used the same lecture slides as in Experiment 2 in [17]. These slides would correspond to lectures 5–7 in the Fall semester 2015. The number of slides were 168, 157 and 144, which corresponded to 57, 54 and 49 securities (the slides were grouped by 3 to define one security). The practice round had 117 slides, which corresponds to 39 securities. The decks of slides can be downloaded here: https://osf.io/6jm9p/. The numbers of the “executed” securities (i.e., which paid out 100 monetary units, corresponding to the ending slide of the lecture) in the Practice and three experimental rounds were 15, 15, 23 and 21, respectively. The slide decks were printed in color such that each security would have 3 slides on one sheet and the number of that security would be marked on each slide with large font.

In contrast to [17], we implemented only three instead of four trading rounds and we reduced the number of slides in Round 1 from 201 to 168, such that the slide deck ends when a certain topic ends. The ending slide was the same as in [17]. We chose to reduce the number of slides in Round 1 in order to adapt it to the available 10-min period and the number of available securities in other rounds. Therefore, the final number of slides in Round 1 was similar to those in other rounds. We used the same trading platform as in [17].

### Appendix A.2. Summary of the Lecturing Style of Prof. Sornette, as Displayed in the Movie

- This movie presents Prof. Didier Sornette’s lecturing style. He prepares more slides than needed and he does not know how much material he will cover.
- Slides that were prepared for a given lecture but were not presented, are presented in a consecutive lecture.
- You will predict the final slide of the lectures that took place in weeks 5–7 of the semester.
- The slides prepared for week 4 will be used in the practice round.
- In weeks 1–3, the professor covered 45, 35 and 30 slides consecutively which corresponds to 69%, 39% and 54% of the available slides.
- There are a few characteristics of the professor’s lecturing style.
- The professor sometimes stops the flow of the lecture to provide a more detailed mathematical derivation of a problem on the blackboard.
- He jumps to a different slide or a topic that either has been shown previously or has not been shown at all.
- Professor Sornette has two lecture ending styles:
  (1) He finishes a topic and ends on the last slide of that topic.
  (2) He finishes a lecture by showing the first slide of the next topic to give an overview on what he will be talking about in the next lecture.
- Some lectures might start a few minutes later due to organisational issues, important announcements or presentation of an assignment.
- Now, you will see samples of the professor’s lecturing style, based on material recording during two consecutive lectures in the Fall 2015.

## Appendix B. Summary of the Self-Reported Measures

A total of 17 participants claimed to have applied a buy-and-hold strategy, 14 classified themselves as using a mean-reverting strategy, 9 reported as trend followers, and 7 people reported “other” strategies. The “other” category included buying cheaply and inflating the prices of the purchased securities to sell at a higher price, buying cheaply and trying to sell expensive and selling securities that were unlikely to pay out the dividend.

The majority of the participants (N=21, 58% of the participants) used the number of slides as the cue for estimating the end slide, while the second most frequent cue was their initial probability estimate (N=18, 50% of the participants). The participants used the bid and ask prices of other traders and the number of topics covered by the professor equally likely (N=15 and 14 consecutively). In the field experiment, substantially more participants would anchor their prediction on the number of the slides covered in the previous rounds but also only twelve participants claimed to be using only one strategy, compared to seven participants (20%) in the laboratory.
