# Supplementary material for: Behavioural Effects and Market Dynamics in Field and Laboratory Experimental Asset Markets
Source: Entropy (Basel). 2020 Oct 20;22(10):1183. doi: 10.3390/e22101183 (PMC7597354; doi:10.3390/e22101183)
Supplement: Supplementary file 1 [file entropy-22-01183-s001.pdf]

Trading Competition (2016)

Instructions to the Experiment

Experimenters:

Prof. Didier Sornette, Dr. Sandra Andraszewicz, MSc Ke Wu, Prof. Ryan Murphy, Dr. Dorsa Sanadgol

Please read the instructions and follow the schedule.

If you have any questions, please raise your hand and ask the assistant in the room.

Dear Participant,

In this experiment, you will trade financial assets with all participants in the experimental room. The financial assets represent slides of Professor Didier Sornette and your task will be to predict on which slide he will finish his lecture because only this slide will pay out a dividend.

You will stay anonymous to other traders for the whole duration of the experiment. No persons from outside of the experimental room have rights to trade on this market and all traders participate in this market for the first time.

There will be three trading sessions. After each session, there will be a short break. Your final performance at the end of the experiment will be calculated as cumulative from all three sessions. Before the three sessions, you will participate in a practice session, which will not count to your final compensation.

Your Compensation

Your performance is based on the cumulative earnings of the three trading sessions. The **top 25%** traders with the highest earnings will receive a bonus of **60 Swiss francs**. The **next 25%** will receive a bonus of **30 Swiss francs**. The remaining 50% of the traders in the rank will not receive any bonus. The bonus will be added to your **base payment of 30 Swiss francs**.

How to Earn the Bonus

In each session, you get an **endowment of 300 Experimental Francs (EFR) and 3 units** of each security. This **loan** has to be **repaid** at the end of the session at the **value of 600 EFR**. At the end of the trading session, only one security pays out a **dividend of 100 EFR**. The stock balance at the end of the session does not contribute to your final earnings, only the cash balance and number of dividends matters. If you generate losses, your balance will be turned to 0. Your balance at the end of each session equals:

$$\boldsymbol{max\{cash\ balance + n * 100\ (dividend)\ -\ 600\ (initial\ endowment), 0\}}$$

Therefore, doing nothing will also earn you nothing. To earn the bonus you have to trade intelligently and/or correctly predict, which security will pay the dividend. Every session will start a new market and earnings or losses are not carried over to the next session.

You can make as many trades as you want, as long as you have enough cash to buy shares and you have enough shares to sell. Short-selling is not allowed.

Which Security Pays a Dividend

Securities correspond to slides of Professor Didier Sornette that he presented in his Financial Markets Risks class in Fall 2015. The professor's teaching style is non-typical in a sense that he prepares more slides than needed. **The security that pays out the dividend corresponds to the final lecture slide that Prof. Sornette presents in his lecture. To receive the dividend you have to correctly predict the final slide of the professor's lecture.** You will receive the stack of slides before the trading session. The final slides of each lecture were recorded in Fall 2015 and they will be announced after every trading session.

Timeline of the Experiment

Step 1 – Professor's Lecturing Style

First, you will see a short movie describing the lecturing style of Prof. Sornette. It will take about 8.5 minutes.

Step 2 – Trading Software Tutorial

Next, you will see a short movie-tutorial on how to use the trading software. It will take about 7.5 minutes.

Step 3 – Practice Session: 5 min trading

In the practice session, you will trade for 5 minutes. Before the trading session starts, you will have 10 minutes to familiarize yourself with the professor's slides and submit your belief. Use the slides that are provided on your desk. This is the time to ask any questions to the experimenters. The experimenters will be present in the room during the practice session. Please, make sure that you understand the software and the procedure before the proceeding to Step 4.

Step 4 – Three Experimental Trading Sessions: 10 min trading

There are three trading sessions that count to your final rank. Each trading session will last 10 minutes and will be proceeded by 10 minutes time to familiarize yourself with the new stack of slides and enter your belief about which slide could be the end slide of the lecture

Step 5 – Debriefing Questionnaire

After the last trading session, you will be automatically re-directed to the website with the questionnaire. Please, enter your trader ID that is provided on your desk and that you used while trading. While you fill out the questionnaire, we will compute the final score on the trading floor.

Practice Session

Each trading session opens at the same time for all participants and will last exactly 5 minutes (10 minutes in the experimental session. The time until the end of the trading will be displayed on your screen.

Login to your account using the login data provided on the table.

At the end of each trading session, the realized number of slides is announced and your account will be credited with your payoff for that session.

In each session, you can monitor your rank.

Before you start trading, you should submit your HONEST and SERIOUS assessment of the probability distribution that the particular security will pay out the dividend.

The Securities

Due to the large number of slides, each security corresponds to three consecutive slides. For your convenience, on each slide, there is a security number and the slides are printed 3 on one page, such that one page corresponds to one security.

There is one “NO-SHOW” security, which would pay 100 EFR in the case where the lecture would be cancelled and the lecture wouldn’t happen.

Practice session will start at 14:35.

Good luck!
